# Modification of the Structure and Linear/Nonlinear Optical Characteristics of PVA/Chitosan Blend through CuO Doping for Eco-Friendly Applications

**DOI:** 10.3390/polym15102391

**Published:** 2023-05-20

**Authors:** Sami S. Alharthi, Ali Badawi

**Affiliations:** Department of Physics, College of Science, Taif University, P.O. Box 11099, Taif 21944, Saudi Arabia

**Keywords:** polyvinyl alcohol/chitosan blend, CuO doping, linear/nonlinear optical, optical bandgap, Wemple-DiDomenico model, eco-friendly applications

## Abstract

The solution casting technique is utilized to fabricate blank and CuO-doped polyvinyl alcohol/chitosan (PVA/CS) blends for eco-friendly applications. The structure and surface morphologies of prepared samples were explored by Fourier transform infrared (FT-IR) spectrophotometry and scanning electron microscopy (SEM), respectively. FT-IR analysis reveals the incorporation of CuO particles within the PVA/CS structure. SEM analysis exposes the well-dispersion of CuO particles in the host medium. The linear/nonlinear optical characteristics were found on the basis of UV-visible-NIR measurements. The transmittance of the PVA/CS decreases upon CuO increasing to 20.0 wt%. The optical bandgap (*E_g dir._*/*E_g ind._*) decreases from 5.38/4.67 eV (blank PVA/CS) to 3.72/3.12 eV (20.0 wt% CuO-PVA/CS). An obvious improvement in the optical constants of the PVA/CS blend is achieved by CuO doping. The Wemple-DiDomenico (WDD) and Sellmeier oscillator models were utilized to examine the CuO role dispersion behavior of the PVA/CS blend. The optical analysis shows clear enrichment of the optical parameters of the PVA/CS host. The novel findings in the current study nominate CuO-doped PVA/CS films for applications in linear/nonlinear optical devices.

## 1. Introduction

Polymeric composites have received great attention because of their effective role in various applications, including the industrial, biological, medical, shielding and entertainment fields [1,2,3,4,5]. Polyvinyl alcohol (PVA), polyvinyl pyrrolidone (PVP), chitosan (CS), carboxymethyl cellulose (CMC) and polyethylene glycol (PEG) possess many attractive features over the rest of polymers, such as non-toxicity, water-solubility, bio-compatibility, eco-friendly and degradability [6,7,8]. Polymeric composites (PCs) are mainly produced by doping small amounts of fillers in a host polymeric matrix for such an application while blending two polymers or more is another scientific trend to yield new polymeric hosts with specific characteristics for updated applications. Particularly, PVA and CS polymers could be blended to produce a novel polymeric host for a lot of applications. PVA possesses high transmittance Vis/NIR regions and a broad bandgap (5.40 eV). Moreover, the hydroxyl groups (—OH) attached to its carbon-chain backbone perform as a hydrogen bonding source that enhances the complexation process [9], while CS, as chitin’s derivative, is the most available polymer that exists in nature [10]. CS could play a dominant role in medical issues because of its unique biocompatibility, antifungal and antimicrobial activities [11]. Mixing PVA and CS produces a PVA/CS polymeric blend to serve as a novel host for various kinds of dopants.

Lots of former works related to PCs are found in the literature. For example, the Heiba research group made great progress using CdS/Mg nanostructures (NPs) on the enhancement of optical characteristics of PVA/CMC. The al-Harthi group proved that the photoluminescent and optical behavior of the PVP/PVA blend could be tailored by incorporating with non-stoichiometric SnS [12]. The microstructure of the PVA/CMC/graphene oxide blend was modified via doping with Fe_3_O_4_ NPs for energy storage issues by Alsulami and Rajeh [13]. Moreover, the optical constants of the PVA/CS blend were enhanced by filling it with graphene/Fe_2_O_3_ for energy storage applications [14]. The Pashameah group concluded that the electrical performance of PVA/CMC was enhanced by MnO_2_ incorporation for optoelectronic applications [15]. The Norouzi research group showed that a PVA/CS blend adapted with TiO_2_/graphene oxide or carbon quantum dots could improve wound healing [16]. Similarly, Venkataprasanna et al. concluded that a CuO-filled CS/PVA/graphene oxide blend could be effectively applied for wound healing [17]. Furthermore, the storage modulus and glass transition temperature of CS were greatly enhanced by Fe_2_O_3_ NPs.

This work focuses on the enhancement of microstructure and linear/nonlinear optical performance of PVA/CS via CuO doping for eco-friendly applications. Copper oxide is preferred as a filler in the PVA/CS blend because of its non-toxicity, abundance, low cost, chemical stability and environmental friendliness. Moreover, its biocompatibility and high antibacterial activities qualify CuO PCs for a lot of medical and daily applications. In addition, CuO’s relatively small optical bandgap (1.2 eV) and large optical absorption coefficient could play a potential role as a filler for LEDs, solar cells, energy storage and other optoelectronic applications. For that, different CuO contents doped in PVA/CS blend were prepared with the solution casting method. The modification in PVA/CS blend structure due to CuO doping was examined by a Fourier transforms infrared (FT-IR) spectrophotometer. The surface morphology was investigated using scanning electron microscopy. Linear/nonlinear optical constants have been investigated based on UV/Vis/NIR data. The obtained investigations reveal the suitability of CuO-doped PVA/CS films for various linear/nonlinear optical applications such as LEDs, fast communications and energy storage devices.

## 2. Methods and Materials

Solution casting technique presented in the literature was carried out to fabricate different contents (0.5, 1.0, 5.0, 10.0 and 20.0 wt%) of CuO-doped PVA/CS polymeric blends. To perform the process, starting sources of PVA (M. W.: 85,000 g∙mol^−1^), chitosan (CS) in powder form (≥75% deacetylated) and copper oxide (CuO; purity > 99.0%) were obtained from Sigma-Aldrich Co. (St. Louis, MO, USA). First, at 70 °C, 7.5 PVA grams were dissolved in 250 mL of double distilled water (DDW) for 4 h. In parallel, 2.5 CS grams were dissolved in acetic acid/DDW/(1:9) at 25 °C for 24 h. Both solutions were mixed for 4 h until a homogenous PVA/CS blend (3:1) was achieved. Next, certain amounts of CuO powder were blended to prepare CuO-doped PVA/CS blends. Afterward, the different CuO-PVA/CS blends were cast in Petri dishes for one day at 55 °C. Next, the samples were peeled out and marked by C_0_ (blank blend) to C_20.0_ (20.0 wt% of CuO-PVA/CS blend). A digital micrometer was used to measure films’ thickness and found 0.18 ± 0.01 mm.

Films’ surface morphology was investigated using a scanning electron microscope (JSE-6390LA, JEOL Ltd., Tokyo, Japan). Absorption bands and structures’ changes were explored at room temperature (RT) using FT-IR (Shimadzu, IRAffinity-1S, Kyoto, Japan) spectrophotometer with the KBr pellets technique. UV-visible-NIR measurements were recorded at RT using a spectrophotometer (JASCO V670, Jasco Corp., Easton, MD, USA). Tauc’s technique was applied to investigate both direct/indirect bandgap (*E_g_*) values as follows [6,18]:

(1) $${\left(\alpha h\nu \right)}^{m}=B(h\nu -E_{g})$$

where $\alpha (=\frac{1}{d}\ln\frac{1}{T}$ [6]), and *d* are optical absorption coefficient and films’ thickness, *B* is constant and *m* is a parameter that may take 2 and 1/2 values for allowed direct/indirect electronic transitions [18,19].

Localized states and created defects’ role in host’s bandgap as a result of CuO doping is investigated via the determination of the Urbach energy (*E_u_*) as follows [13]:

(2) $$\alpha =\alpha_{0}\exp(h\nu /E_{u})$$

where *α*_0_ is a constant.

The refractive index (*n*), extinction coefficient (*K*), and optical conductivity (*σ_opt._*) in UV/Vis/NIR regions were calculated as

(3) $$n=\left(\frac{1+R}{1-R}\right)+{\left[\frac{4R}{{\left(1-R\right)}^{2}}-K^{2}\right]}^{1/2}$$

(4) $$K=\frac{\alpha \lambda }{4\pi }$$

(5) $$\sigma_{opt.}=\frac{\alpha nC}{4\pi }$$

where *C* is light speed, *R* is reflectance, and *λ* is photons/wavelength.

The dielectric permittivity constants (real *ε_r_*, imaginary *ε_i_*) and surface/volume energy loss functions (SELF/VELF) were also determined from [20,21]:

(6) $$\varepsilon_{r}=n^{2}-K^{2}$$

(7) $$\varepsilon_{i}=2nK$$

(8) $$SELF=\frac{\varepsilon_{i}}{{\left(\varepsilon_{r}+1\right)}^{2}+\varepsilon_{i}^{2}}$$

(9) $$VELF=\frac{\varepsilon_{i}}{\varepsilon_{r}^{2}+\varepsilon_{i}^{2}}$$

Moreover, Wemple-DiDomenico (WDD) model was followed to examine *n* dispersion [22], whereas Sellmeier oscillator relations were applied to investigate the rest of the optical parameters as infinite refractive index (*n*_ꝏ_), average oscillator strength (*S*_0_), average inter-band oscillator wavelength (*λ*_0_), infinite dielectric parameter (*ε*_ꝏ_), lattice dielectric parameter (*ε_L_*) and free carrier concentration/effective mass (*N*/m*) as [23,24]:

(10) $$n^{2}=1+\frac{E_{d}E_{0}}{E_{0}^{2}-{\left(h\nu \right)}^{2}}$$

(11) $$\frac{n_{\infty }^{2}-1}{n^{2}-1}=1-{\left(\frac{\lambda_{0}}{\lambda }\right)}^{2}$$

(12) $${\left(n^{2}-1\right)}^{-1}=\frac{1-{\left(\frac{\lambda_{0}}{\lambda }\right)}^{2}}{S_{0}\lambda_{0}^{2}}$$

(13) $$S_{0}=\frac{n_{\infty }^{2}-1}{\lambda_{0}^{2}}$$

(14) $$\varepsilon_{\infty }=n_{\infty }^{2}$$

(15) $$\varepsilon_{r}=n^{2}=\varepsilon_{L}-\frac{e^{2}}{4\pi^{2}C^{2}\varepsilon_{0}}\frac{N}{m^{*}}\lambda^{2}$$

where *e* is free electron charge, and *ε*_0_ is space dielectric constant.

The linear first-order susceptibility (*χ*^(1)^), nonlinear third-order susceptibility (*χ*^(3)^) and nonlinear refractive index (*n*_2_) were investigated as [25,26]

(16) $$\chi^{\left(1\right)}=\frac{n^{2}-1}{4\pi }$$

(17) $$\chi^{\left(3\right)}=1.7\times 10^{-10}{\left(\chi^{\left(1\right)}\right)}^{4}$$

(18) $$n_{2}=\frac{12\pi }{n}\chi^{\left(3\right)}$$

## 3. Results and Discussion

### 3.1. Morphological Analysis

**Figure 1:**
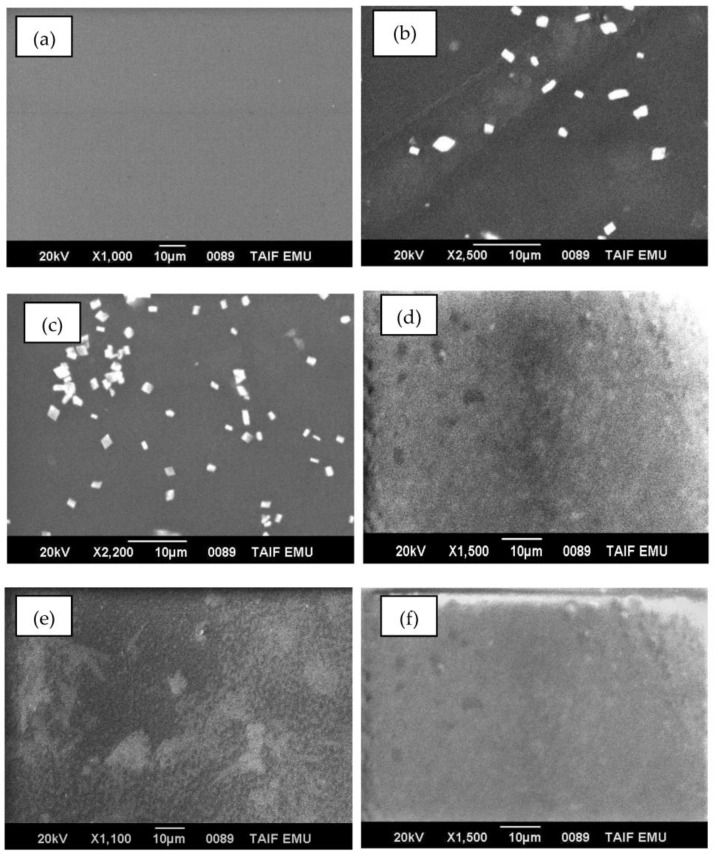
SEM micrograms of (**a**) blank and (**b**–**f**) different (0.5 to 20 wt%) CuO-PVA/CS films.

Films’ surface morphologies were captured by a scanning electron microscope (SEM). Figure 1a–f illustrates SEM micrograms of the blank and different (0.5 to 20 wt%) CuO-PVA/CS films, respectively. The SEM microgram of the blank film is spot-free with a smooth surface (Figure 1a), whereas distinguishable bright spots related to the CuO granules are clearly noticed in SEM micrographs of 0.5 and 1.0 wt% of CuO-PVA/CS films. These spots become denser, closer and more compact as the CuO concentration is increased to 20 wt%.

### 3.2. FT-IR Analysis

**Figure 2:**
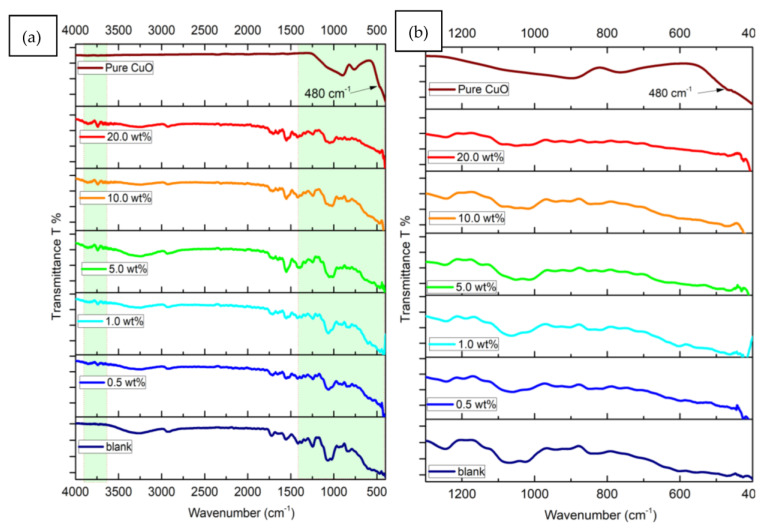
FT-IR spectra (**a**) full wavelength range and (**b**) 1300–400 cm^−1^ of blank and CuO-PVA/CS films.

Figure 2a,b depicts FT-IR transmittance spectra of blank and different CuO-PVA/CS films in the 400 to 4000 cm^−1^ range, as demonstrated by plots, clear variations in intensity and sites of dominant absorption bands of doped samples with respect to the blank one. These changes confirm the interactions between the CuO molecules with the structure of the host PVA/CS matrix. This interaction mainly takes place by replacing the OH groups in the host structure with that of the CuO ones [27]. Relative to FT-IR spectra of blank PVA/CS film and pure CuO material, the main absorption bands and vibrations are recorded (Table 1). Similar FT-IR performance is noticed in the CuO-PVA/CS films with clear intensity variations and slight location shifts with broadening in the absorption bands. These changes are pronounced in the regions 3900–3600 cm^−1^ and 1300–400 cm^−1^ as background shadows in Figure 1a, whereas the absorption bands correspond to the Cu-O bonds may overlap with those of the host matrix at the 1300–400 cm^−1^ region, as shown in Figure 1b. Our findings reveal the complete incorporation of CuO and the host medium. The same trends are reported in the literature [27,28,29].

### 3.3. UV/Vis/NIR Investigations

**Figure 3:**
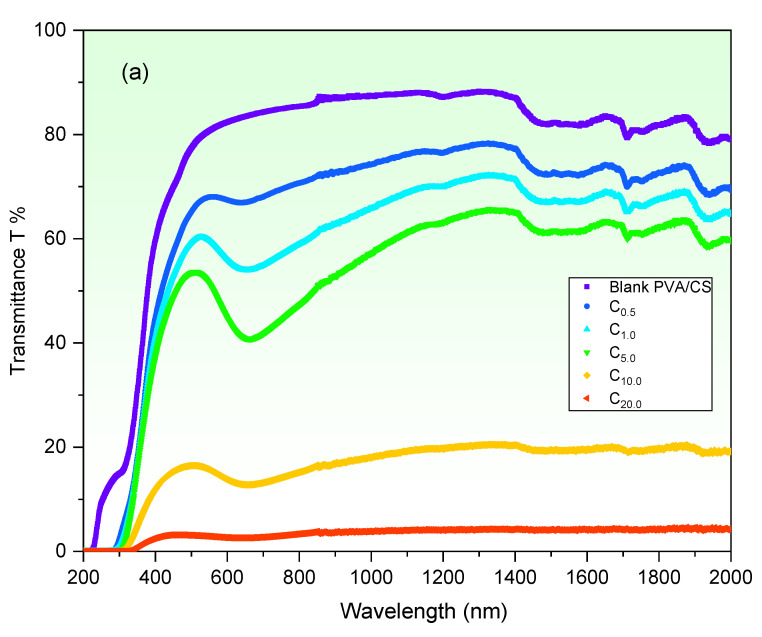

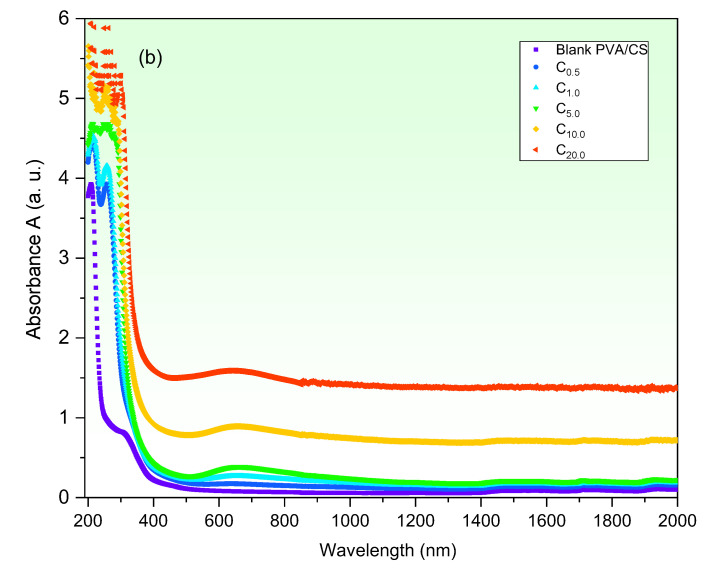
(**a**) Wavelength dependence of the transmittance (T) and (**b**) absorbance (A) spectra of blank and different CuO-PVA/CS films.

**Figure 4:**
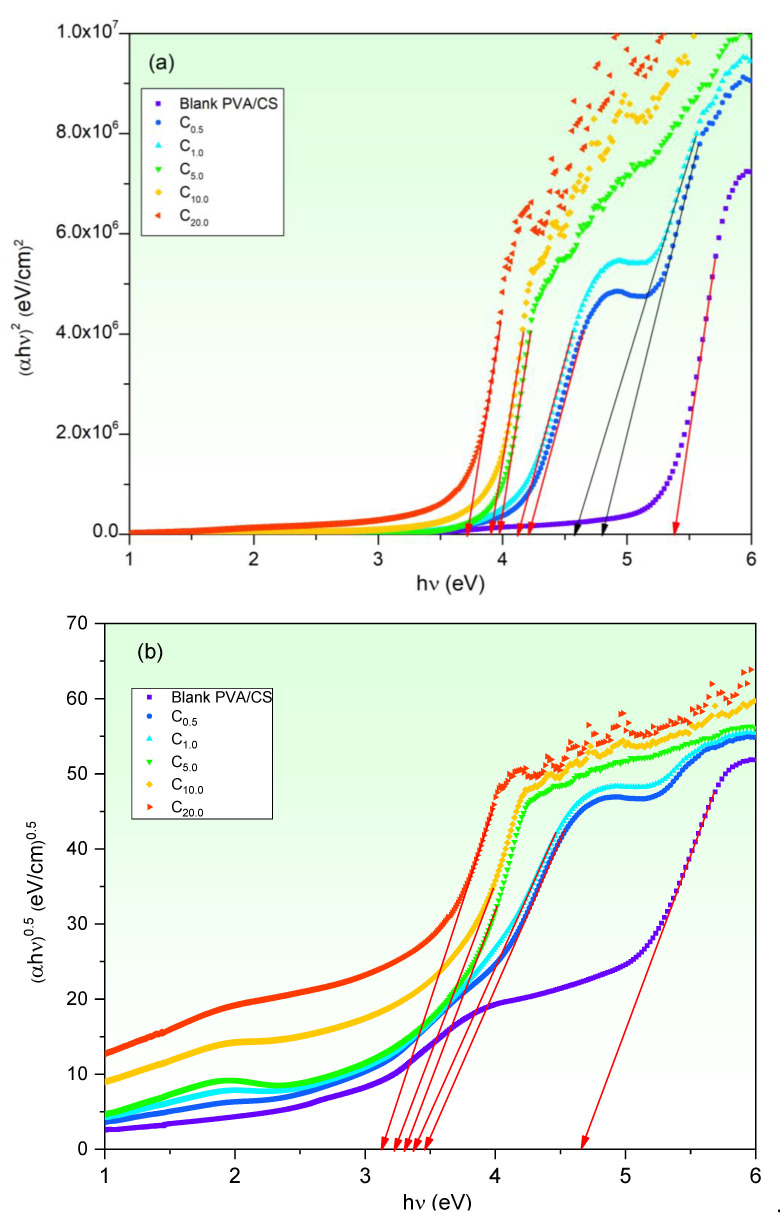
Tauc’s plots (**a**) direct and (**b**) indirect status of CuO-PVA/CS films.

The effect of CuO concentration on the optical parameters of the PVA/CS blend has been explored on the basis of the UV/Vis/NIR measurements. The wavelength dependence of the transmittance (T) and absorbance (A) of blank and different CuO contents filled PVA/CS blends are presented in Figure 3a,b, respectively. It is noticed that at any certain *λ*, T decreases in visible-NIR regions as the CuO content is increased from 0 to 20 wt%. For example, the T of the blank PVA/CS film is more than 80% in the visible region, while it decreases to about 3% for 20 wt% of CuO-PVA/CS film in the same region. Moreover, as the CuO content is increased from 0 to 20 wt%, the UV cut-off edge is red-shifted to longer wavelengths from 225 nm to 358 nm. This valuable result nominates the possible role of CuO-PVA/CS films in UV-shielding applications. In contrast, the absorption increases due to the increase of CuO contents. In addition, clear redshifts in the absorption edges are noticed. Furthermore, two absorption peaks at 211 nm and 258 nm are detected in all absorption spectra that correspond to the PVA electronic π → π^*^ transitions [42], whereas the absorption edge detected at 324 nm refers to the electronic n → π^*^ transitions [43]. The decrease in the optical transmittance and hence increment in the absorption amounts due to CuO doping is attributed to the increase in defects (shown below), which leads to a decrease in the optical band gap of the PVA/CS blend, as discussed in Figure 4.

Based on Tauc’s equation (Equation (1)), direct/indirect optical bandgap (*E_g dir._/E_g ind._*) of blank and CuO-PVA/CS films has been obtained from (*αhν*)^2^ and (*αhν*)^1/2^ curves vs. *hν*, respectively, as depicted (Figure 4). The *x*-axis intercepts of extrapolated linear parts of these curves to *hν* = 0 equal *E_g_* values as listed in Table 2. The obtained *E_g dir._/E_g ind._* values of blank PVA/CS film are 5.38 eV and 4.67 eV. These values are well-consistent with the reported ones [34,44]. The *E_g dir._/E_g ind._* values of CuO-PVA/CS films decrease to 3.72 eV and 3.12 eV as CuO concentration is upraised to 20 wt%. Moreover, it is clear that both 0.5 wt% and 1.0 wt% CuO-PVA/CS films possess second bandgap values of 4.79 eV and 4.57 eV, respectively, as illustrated in Figure 4a. This finding reveals that the absorption happens as a result of charge transfer between two different energy levels. The first transition occurs between the molecular orbits of the host matrix, while the other electronic transition takes place between the created energy state due to CuO particles and those of the host matrix. Similar findings were recorded in previous works [21,45]. So, the *E_g_* narrowing mainly results due to localized states and defects created between the highest occupied molecular orbital (HOMO) and lowest unoccupied molecular orbital (LUMO) of the PVA/CS blend due to CuO doping [42,46]. Similarly, Heiba et al. concluded that 4 wt% of Cd_0.9_Mg_0.1_S nanofillers led to a reduction in the *E_g_* of PVA/CMC blend from 5.4 eV to 5.02 eV [44]. Additionally, the *E_g_* of the PVA/CMC/GO blend was reduced to 3.34 eV using 1.0 wt% of Fe_3_O_4_ doping [13]. Formerly, we modified the optical bandgap of PVA/Gr from 5.38 eV to 4.78 eV by Fe_2_O_3_ doping [47].

**Figure 5:**
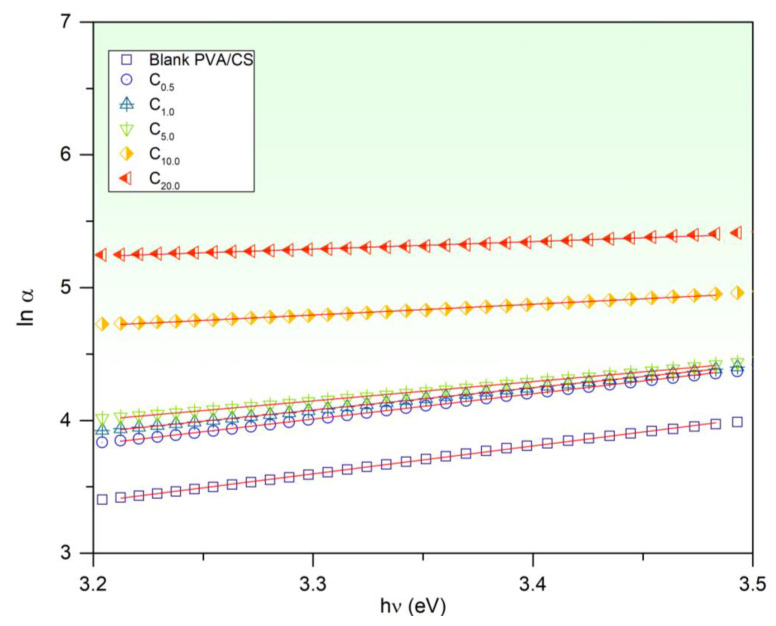
*lnα* vs. *hν* of CuO-PVA/CS films.

The defects and localized energy states created in CuO-PVA/CS films could be proved by investigating the Urbach energy (*E_u_*) (Equation (2)). It shows the exponential dependence of the absorption coefficient and photons energy (*hν*). *E_u_* is estimated (Table 2) by plotting *lnα* vs. *hν,* as illustrated in Figure 5. It was noticed that *E_u_* grows from 0.48 eV (blank PVA/CS) to 1.79 eV (20 wt% CuO-PVA/CS). The increase in *E_u_* indicates the growth of localized states and defects that works as trapping centers in the forbidden region of the PVA/CS host [48]. Similar evidence is reported in the literature [13,25,49]. As an original result, the optical bandgap of PVA/CS is tailored by CuO doping for a lot of optical and environmental applications.

**Figure 6:**
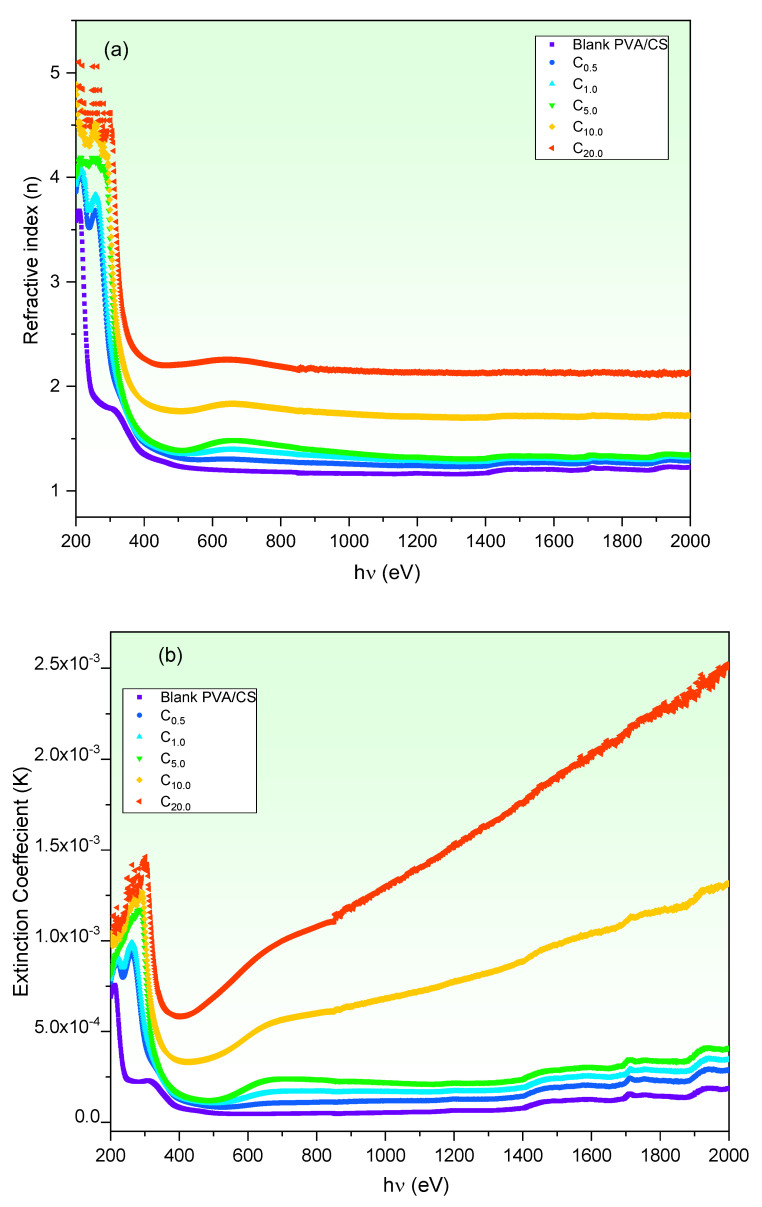
(**a**) *n* and (**b**) *K* vs. wavelength of CuO-PVA/CS films.

The optical performance of such material is mainly established by investigating the refractive index (n* = n-i*K*) to dictate its applications. The real (n) and imaginary (*K*) parts describe the dispersion behavior of the electromagnetic wave within the material. Both *n* and *K* at the swept wavelength (*λ*) are calculated using Equations (3) and (4), respectively. The wavelength dependence of *n* and *K* of blank and CuO-PVA/CS films are illustrated in Figure 6a,b, respectively. According to Figure 6a, it is noted that *n* follows the absorbance performance (Figure 3b). In other words, *n* decreases steeply upon raising *λ* in the UV region, whereas it remains semi-steady in Vis/NIR regions. Moreover, it is seen that the *n* of PVA/CS is enhanced as a result of CuO doping, which proposes it for updated applications in optical and optoelectronic devices. For instance, *n* increases from 1.2 (blank PVA/CS) to 2.25 (20 wt% CuO-PVA/CS) at 650 nm. The improvement in the *n* value refers to the growth in the films’ density and intermolecular bonds due to CuO doping [27,50,51], whereas *n* remains quasi-steadily in low photons energy due to films’ restricted absorbance in this region, whereas, according to Figure 6b, *K* declines with increasing *λ* in the UV region, whereas it increases gradually in visible-NIR regions. Furthermore, *K* increases as the CuO content is increased. These findings could be understood on the basis of the increment of the dispersion as a result of the reflectance increase due to defects’ growth [51].

**Figure 7:**
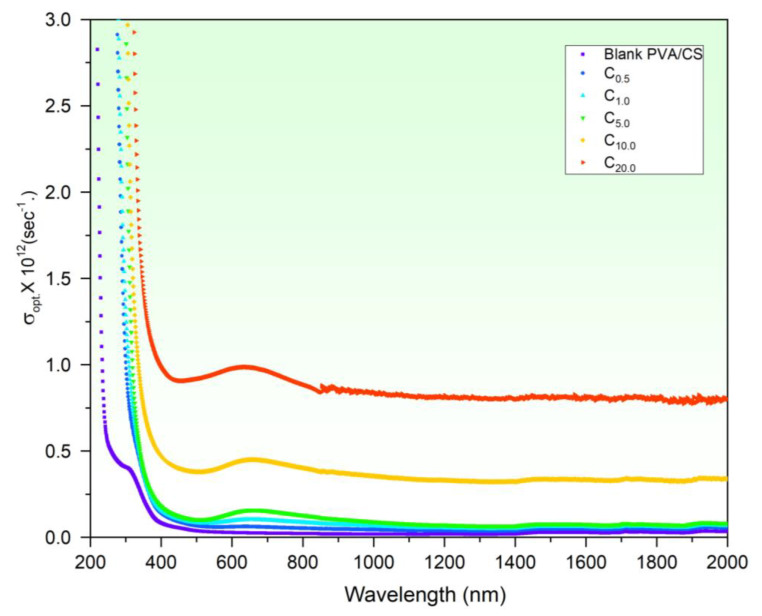
Optical conductivity vs. wavelength of CuO-PVA/CS films.

Based on the absorption coefficient α and *n*, *σ_opt._* of CuO-PVA/CS samples was determined (Equation (5)) and illustrated in Figure 7. It is observed that *σ_opt._* of the films behaves in a similar way to the optical absorbance (Figure 3b). As *λ* is red-shifted to longer values in the UV region, *σ_opt._* decreases steeply, while it behaves steadily in the Vis/NIR regions. In contrast, *σ_opt._* increases upon increasing the CuO content in the PVA/CS host. For example, at 650 nm, *σ_opt._* enhances from 2.56 × 10^10^ s^−1^ (blank PVA/CS) to 9.85 × 10^10^ s^−1^ (20 wt CuO-PVA/CS). The *σ_opt._* enhancement is understood on the basis of the increment in created electrons as a result of the absorption increase of the incident photons [27,52]. The increase in absorption is also reinforced by the growth in the defects, as discussed in E_u_ findings. These findings are very consistent with reported data [21,53,54]. Shamekh et al. proved that *σ_opt._* of PVA was pronouncedly enhanced by MgO doping.

**Figure 8:**
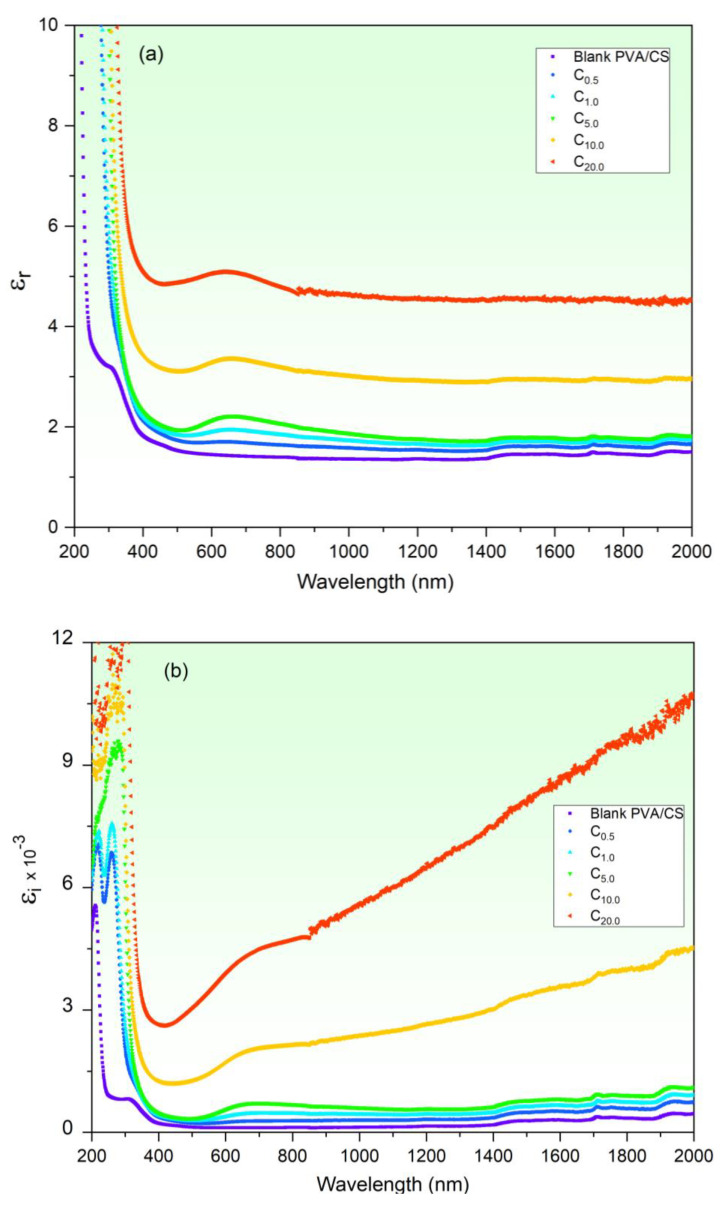

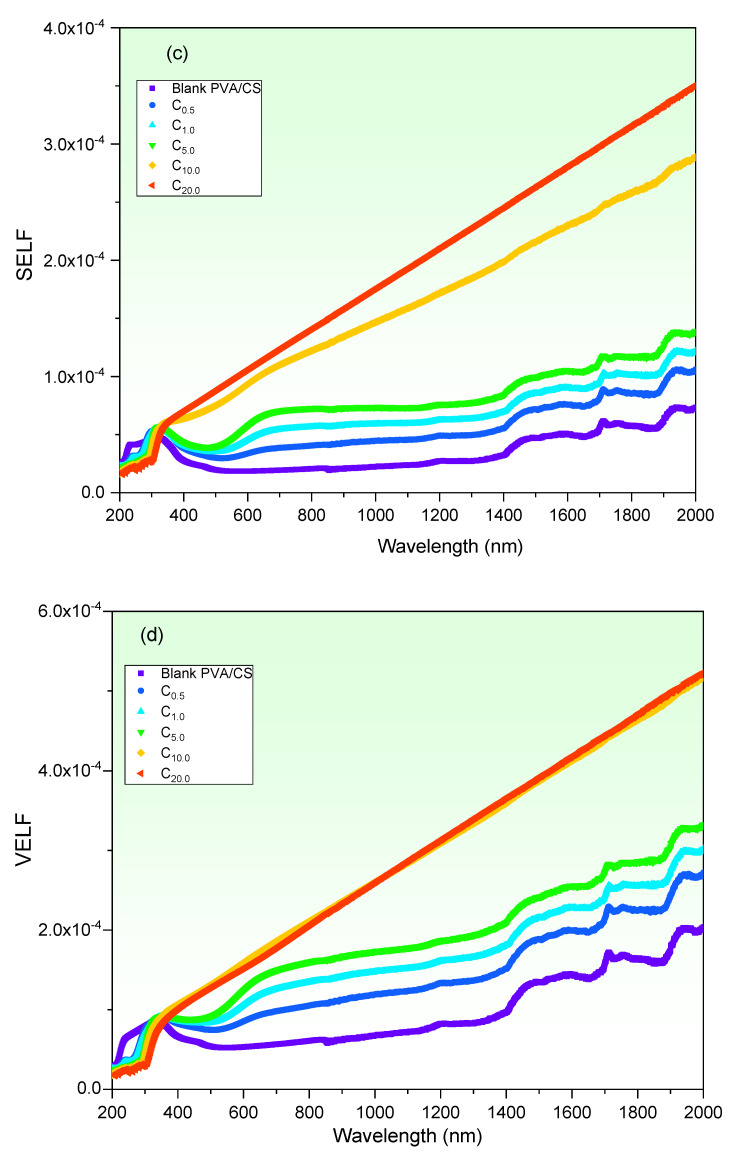
(**a**) Real, (**b**) imaginary dielectric constant. (**c**) SELF and (**d**) VELF of CuO-PVA/CS films.

The dielectric parameters (*ε_r_* and *ε_i_*), together with the surface/volume energy loss functions (SELF/VELF) of the blank and CuO-PVA/CS films, have been determined. These constants are investigated to nominate their possible participation in many fields as superconductors and energy storage devices. *ε_r_* associates with traveling wave dispersions within such material, while *ε_i_* relates to the dissipated energy rate through their propagation [21]. *ε_r_*, *ε_i_*, SELF and VELF were calculated by Equations (6) to (9) and presented in Figure 8a–d, respectively. According to *ε_r_* spectra (Figure 8a), it follows the refractive index *n* performance. *ε_r_* decreases steeply as *λ* is red-shifted in the UV region, whereas it remains semi-constant in the visible-NIR regions. Furthermore, *ε_r_* rises as CuO content is increased to 20 wt%. For example, *ε_r_* enhanced from 1.43 (blank PVA/CS) to 5.08 (20 wt% CuO-PVA/CS) at *λ* = 650 nm. The enhancement in ε_r_ results due to the increase in the dispersion as a result of a defects increase (Urbach energy findings). On the other hand, the imaginary part *ε_i_* of the film performs similarly to the extinction coefficient *K* (Figure 6b). *ε_i_* decreases greatly as *λ* increases in the UV region, while it increases slowly in Vis/NIR region for small CuO contents (≤5 wt%) and increases pronouncedly for the high CuO contents (10 and 20 wt%). Moreover, *ε_i_* increases as the CuO content is raised. This behavior refers to polarization and dipole motion fluctuations [26,55,56]. Similar findings are reported in the literature [26,57,58]. Moreover, it is noticed that SELF and VELF spectra perform in a similar way. Both SELF and VELF values increase noticeably as *λ* is red-shifted to longer wavelengths in the Vis/NIR regions. Moreover, it is noted that at any *λ*, the VELF value is larger than the SELF value, which indicates that the energy loss by the traveling electrons within the films due to the doped CuO particles is larger than those traveling on their surfaces. In addition, both SELF and VELF increased upon increasing the CuO contents. This increment in SELF and VELF refers to growth in vacant energy levels generated in the host band gap [59]. Similar behavior is noticed El-naggar et al. [26]. They showed SELF and VELF increase of the PVA/PVP upon increasing SnS_2_/Fe concentration.

**Figure 9:**
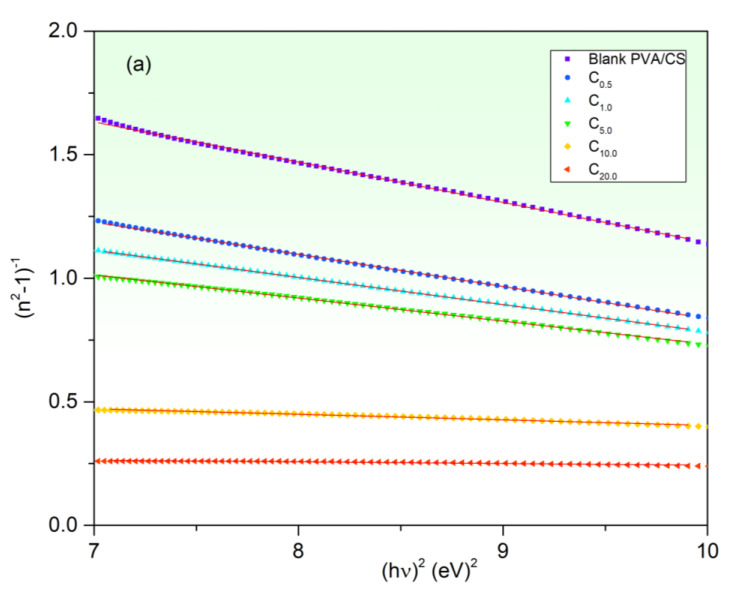

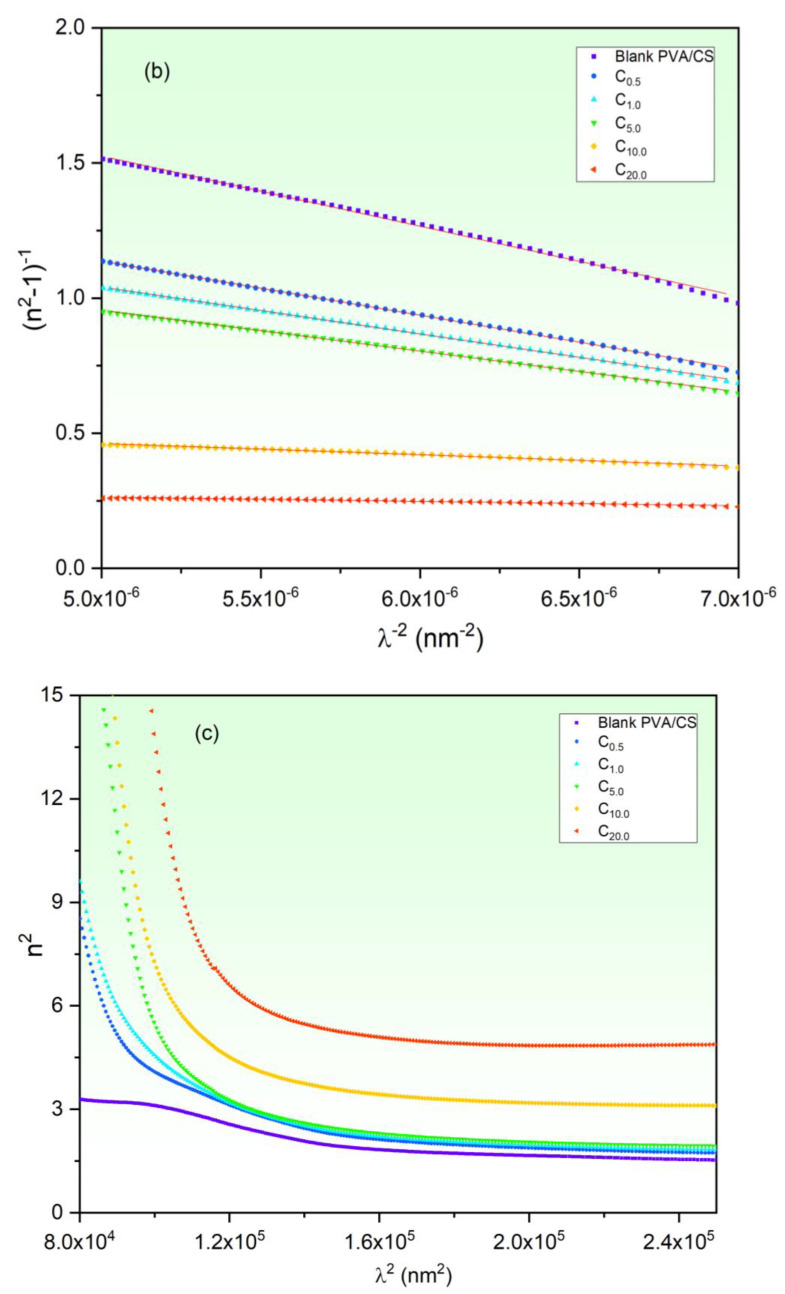
(**a**) (*n*^2^−1)^−1^ vs. (*hν*)^2^, (**b**) (*n*^2^−1)^−1^ curves vs. *λ*^−2^ and (**c**) *n*^2^ curves vs. *λ*^2^ of CuO-PVA/CS films.

Moreover, the dispersion parameters of the blank and CuO-PVA/CS films are examined by a single oscillator model (WDD model; Equation (10)) in the normal dispersion region. Investigating *E_o_* and *E_d_* are essential parameters to nominate the applications of the prepared films in communication systems and spectra analysis devices [21]. The values of *E_o_* and *E_d_* are found from (*n*^2^ − 1)^−1^ plots vs. (*hν*)^2^ as depicted in Figure 9a, where the slopes equal −1/(*E*_0_*E_d_*) and the intersections equal E_0_/E_d_. Table 3 includes E_o_ and E_d_ values. Both *E_o_* and *E_d_* values increase upon increasing the CuO content in the host PVA/CS. This increase in the dispersion energies refers to the increase in the optical transition strength of the system bonds [60].

Furthermore, the infinite refractive index (*n_ꝏ_*), the infinite dielectric constant (ε_ꝏ_), and the average oscillator strength (*S*_0_)) of the blank and CuO-PVA/CS films are determined on the basis of the Sellmeier oscillator relations (Equations (11) to (15)). By plotting (*n*^2^ − 1)^−1^ vs. *λ*^−2^ (Figure 9b) and equating the slopes with 1/*S*_0_ and the intersections with $1/S_{0}\lambda_{0}^{2}$, the values of *λ*_0_, *n_ꝏ_*, *S*_0_ and ε_ꝏ_ are obtained and listed in Table 3. While N/m* and ε_L_ are obtained by plotting *n*^2^ vs. *λ*^2^ (Figure 9c), where the slopes $\left(=\frac{e^{2}}{4\pi^{2}C^{2}\varepsilon_{0}}\frac{N}{m^{*}}\right)$ and intersections (=*ε_L_*) as listed in Table 3. It is obvious that all optical behaviors of the PVA/CS blend are altered with CuO doping. For example, *ε_ꝏ_* of the blank PVA/CS film is greatly enhanced from 1.35 to 3.94 (20 wt% CuO-PVA/CS film). The enhancement in *ε_L_* and ε_ꝏ_ refers to the dispersion lattice vibrations as a result of CuO particles [37]. Similar *ε_L_* and *ε_ꝏ_* findings related to polystyrene filled with manganese (III) chloride were found by Al-Muntaser et al. [37], while N/m* of the blank PVA/CS film is duplicated due to 20 wt% of CuO doping. This result is reasonable as a result of the increment of the free carriers due to CuO doping [21]. Our results are compatible with the literature [61,62].

**Figure 10:**
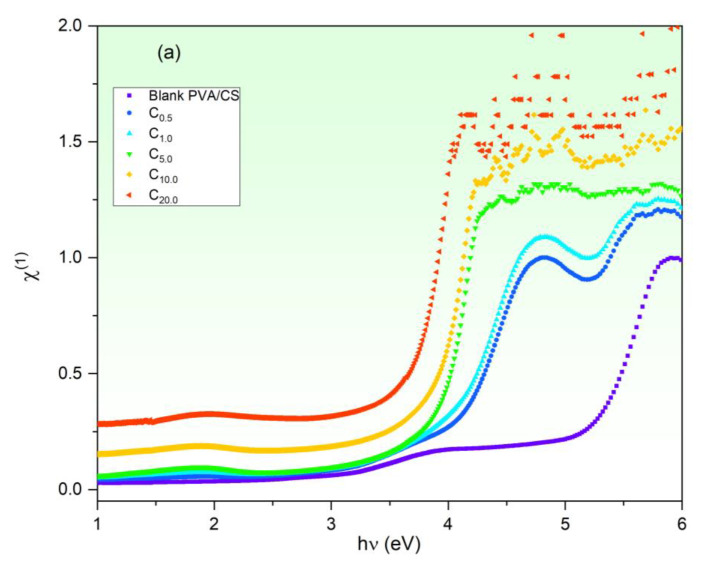

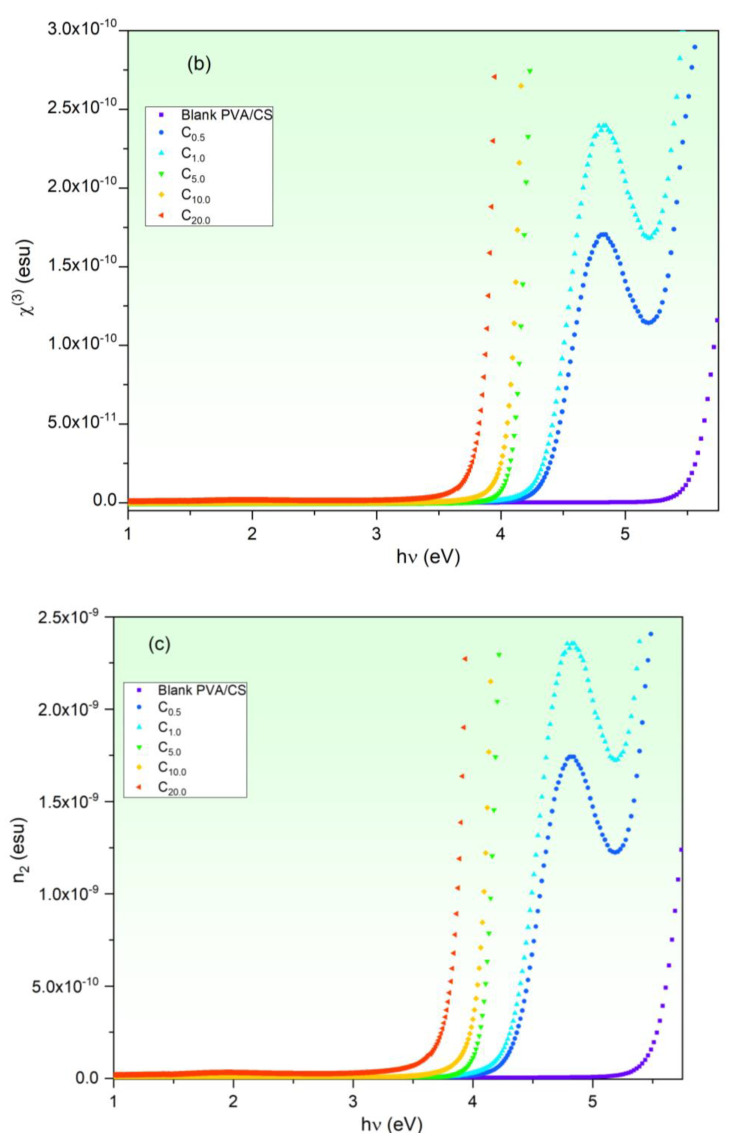
(**a**) First-order susceptibility (χ^(1)^), (**b**) third-order susceptibility (χ^(3)^) and (**c**) nonlinear refractive index (*n*_2_) of CuO-PVA/CS films.

The nonlinear optical behavior of blank and CuO-PVA/CS samples is explored to recommend their probable applications in nonlinear optical devices. Optical materials with the optical nonlinearity character play an effective role in many applications such as ultrafast lasing switching, frequency converters and telecommunications [21,63]. The nonlinear optical response arises because of the nonlinear polarization that occurs owing to intense electromagnetic wave exposure [21,64,65]. Based on Equations (16) to (18), χ^(1)^, χ^(3)^ and n_2_ are calculated and presented in Figure 10a–c, respectively. It is noted that χ^(1)^, χ^(3)^ and n_2_ behave semi-steadily in the Vis/NIR regions, whereas they rise rapidly upon increasing *hν* in the UV region. In addition, as the CuO content is increased to 20 wt%, χ^(1)^, χ^(3)^ and n_2_ increase noticeably. For instance, at 4.0 eV, χ^(1)^ of the blank PVA/CS film is enhanced from 0.17 esu to 1.36 esu via 20 wt% CuO doping, while χ^(3)^ and n_2_ of the blank film are enhanced about by three-order of magnitude at the same incident photons energy. These findings are compatible with previous works [25,26,64]. For example, the Ali group found that the nonlinear optical constant of PVA was enhanced pronouncedly by fullerene doping [25]. The obtained nonlinear optical findings of the CuO-PVA/CS films in this study nominate their applications in nonlinear optical devices [65].

## 4. Conclusions

The solution casting method was followed to fabricate blank and CuO doped in polyvinyl alcohol/chitosan (PVA/CS) blends. The effect of CuO concentrations (0, 0.5, 1.0, 5.0, 10.0 and 20.0 wt%) on PVA/CS structure and linear/nonlinear optical characteristics is discussed in detail. Scanning electron microscope examinations disclose obvious changes in the surface morphologies of PVA/CS film owing to CuO doping. FT-IR measurements prove noticeable modifications in PVA/CS structure due to CuO doping. Noticeable modifications in absorption band’s locations and intensities of CuO-PVA/CS films as compared with the blank one. The linear/nonlinear optical parameters were discussed. The transmittance of the PVA/CS blend reduces as a result of CuO increasing to 20.0 wt%. The optical bandgap (E_g dir._/E_g ind._) decreases from 5.38/4.67 eV (blank PVA/CS) to 3.72/3.12 eV (20.0 wt% CuO-PVA/CS). This decrease in the optical bandgap is interpreted in terms of defects and created states, as verified by Urbach energy investigations. The refractive index, optical conductivity and dielectric constants of PVA/CS are clearly enhanced due to CuO doping, which nominates it for updated applications in optoelectronic devices. The CuO doping role in the dispersion performance of PVA/CS has been investigated using a single oscillator and Sellmeier oscillator relations. For instance, the infinite dielectric constant is greatly enhanced from 1.35 (blank PVA/CS) to 3.94 (20 wt% CuO-PVA/CS film), whereas the concentration of free carriers/effective mass of blank PVA/CS film is duplicated. The nonlinear optical parameters of PVA/CS are also enhanced via CuO doping. χ^(3)^ and n_2_ are improved by about three orders-of-magnitude at 4.0 eV incident photons energy. These novel findings nominate CuO-PVA/CS films for updated optical applications.

## Figures and Tables

**Table 1:** FT-IR absorption bands and bonds’ vibration.

Wavenumber Site (cm^−1^)	Bond Vibration	References
3280	O—H stretching	[30,31]
2915	C—H asymmetric stretching	[13,32]
1724	C=O stretching	[30,33]
1552	O—H and C—H bending	[34]
1423	C—H bending	[33,35]
1251	C—H wagging	[33,34]
1069	C—O bending	[27,28]
832	C—C stretching	[34,36]
435	O—H wagging, C—C bending and CuO	[29,33,37]
891	Cu—O—Cu stretching	[38,39]
762	Cu—O stretching	[38,40]
480	Cu—O stretching	[41]

**Table 2:** Direct/indirect bandgap, Urbach energy and refractive index of CuO-PVA/CS films.

CuO wt%	Direct *E_g dir._* (eV)	Indirect *E_g ind._* (eV)	*Eu* (eV)	*n* @ 650 nm
0	5.38	4.67	0.48	1.20
0.5	4.21	3.45	0.52	1.31
1.0	4.13	3.38	0.60	1.39
5.0	3.97	3.29	0.69	1.49
10.0	3.90	3.22	1.24	1.83
20.0	3.72	3.12	1.79	2.25

**Table 3:** Dispersive parameters of CuO-PVA/CS films.

CuO wt%	*E_d_* (eV)	*E*_0_ (eV)	*n_ꝏ_*	*λ*_0_ (nm)	*S*_0_ (m^−2^)	ε_ꝏ_	ε_L_	(N/m*) × 10^57^ (kg^−1^.m^−3^)
Blank PVA/CS	1.49	4.14	1.16	302.8	3.85 × 10^12^	1.35	2.33	4.01
0.5	1.89	4.06	1.21	306.2	5.01 × 10^12^	1.47	2.79	5.37
1.0	2.20	4.14	1.24	301.7	5.78 × 10^12^	1.53	2.85	5.21
5.0	2.54	4.24	1.26	297.2	6.62 × 10^12^	1.58	2.92	5.09
10.0	7.27	4.58	1.55	243.2	2.38 × 10^13^	2.41	4.52	7.88
20.0	21.13	6.76	1.98	216.9	6.25 × 10^13^	3.94	5.37	8.85

## Data Availability

The data are available on reasonable request from the corresponding author.
